# Orofacial neuropathic pain mouse model induced by Trigeminal Inflammatory Compression (TIC) of the infraorbital nerve

**DOI:** 10.1186/1756-6606-5-44

**Published:** 2012-12-28

**Authors:** Fei Ma, Liping Zhang, Danielle Lyons, Karin N Westlund

**Affiliations:** 1 Department of Physiology MS-508, College of Medicine, University of Kentucky, Lexington, KY, 40536-0298, USA

**Keywords:** Orofacial neuropathic pain, Infraorbital nerve, Inflammation, Nerve compression, Chromic gut suture, Mechanical allodynia, Trigeminal ganglia, Trigeminal nucleus, Mice, Hypersensitivy, Tic douloureux

## Abstract

**Background:**

Trigeminal neuropathic pain attacks can be excruciating for patients, even after being lightly touched. Although there are rodent trigeminal nerve research models to study orofacial pain, few models have been applied to studies in mice. A mouse trigeminal inflammatory compression (TIC) model is introduced here which successfully and reliably promotes vibrissal whisker pad hypersensitivity.

**Results:**

The chronic orofacial neuropathic pain model is induced after surgical placement of chromic gut suture in the infraorbital nerve fissure in the maxillary bone. Slight compression and chemical effects of the chromic gut suture on the portion of the infraorbital nerve contacted cause mild nerve trauma. Nerve edema is observed in the contacting infraorbital nerve bundle as well as macrophage infiltration in the trigeminal ganglia. Centrally in the spinal trigeminal nucleus, increased immunoreactivity for an activated microglial marker is evident (OX42, postoperative day 70). Mechanical thresholds of the affected whisker pad are significantly decreased on day 3 after chromic gut suture placement, persisting at least 10 weeks. The mechanical allodynia is reversed by suppression of microglial activation. Cold allodynia was detected at 4 weeks.

**Conclusions:**

A simple, effective, and reproducible chronic mouse model mimicking clinical orofacial neuropathic pain (Type 2) is induced by placing chromic gut suture between the infraorbital nerve and the maxillary bone. The method produces mild inflammatory compression with significant continuous mechanical allodynia persisting at least 10 weeks and cold allodynia measureable at 4 weeks.

## Background

Chronic orofacial pain regardless of the origin along the trigeminal nerve is particularly debilitating and often refractory to treatment. While the cause of trigeminal neuropathic pain is often unknown, nerve trauma, compression and/or demyelination are the most probable causes [1]. Using two loose chromic gut suture ligatures applied to the infraorbital branch of the trigeminal nerve to promote orofacial hypersensitivity, Vos et al. [2] first adapted the widely used method referred to as the chronic constriction injury (CCI) model by Bennett and Xie [3] which is typically applied to the sciatic nerve in rats. Since that time several models have been developed for rodents using the inferior alveolar, mental, or the infraorbital as target nerves for injury models in orofacial pain studies. However, these nerves and their whisker pad receptive field testing areas are of much smaller size in mice compared to rats. Unlike the nerve constriction surgery in rats, the small operating space and abundant blood supply in the facial area make the mouse infraorbital nerve injury model particularly challenging. Thus, effective mouse models of chronic orofacial neuropathic pain are limited. The method reported here provides a stable orofacial neuropathic pain model which better mimics clinical chronic orofacial neuropathic pain.

Several other nerve injury methods have been applied to the infraorbital nerve in mice. While a constrictive nerve injury is reported for mice by Luiz et al. [4], ligations are formed with more pliable silk suture rather than chromic gut suture. In this model the appearance of thermal hypersensitivity occurs 3 weeks post-surgery. Partial (one third to one half) infraorbital nerve tight ligation with silk suture provides mechanical allodynia that does not recover in 25 days while face grooming diminishes within 7 days [5]. Similar functional outcome is reported with partial transection of the infraorbital nerve in another study [6]. In another previous report, sensation impairment persists more than 10 weeks after tight ligation of the mental nerve in mice, however, the behavioral test describes grabbing the mice from the back to take pain threshold measurements in this unnatural posture [7].

Ligating the infraorbital nerve with chromic gut sutures in rats produces mechanical allodynia in response to von Frey filament stimulus and facial grooming that persists over three months as described by Vos et al. [2], compared to the one month duration of the hyperalgesia with use of silk ligature in mice [4]. We have developed a mouse model of chronic orofacial neuropathic pain using chromic gut suture to inflame the infraorbital nerve. The method is adapted from that described by Maves et al. [8], who aligned different lengths of chromic gut suture along the sciatic nerve in rats and found “dose-dependent” hyperalgesia and allodynia develop. To induce the TIC model described here, chromic gut suture is placed beneath the infraorbital nerve in the infraorbital fissure of the maxillary bone. Blood circulation through the superficial epineurial vasculature is preserved, but the suture causes mild nerve compression. The chromic salts and pyrogallol released from chromic gut suture cause a change in the chemical milieu locally on one side of the nerve. The partial damage to the nerve and inflammation initiates mechanical allodynia that is evident in the mouse whisker pad throughout a 10 week experimental time course and cold allodynia at a 4 week time point tested. Data are also provided documenting reversal of the mechanical allodynia with several drugs that have been shown to effectively alleviate peripheral nerve injury induced neuropathic pain [9-11], including microglial activation inhibitor, minocycline; P2X7 antagonist, A438079; and p38 inhibitor, SB203580. The simplified TIC model produced by surgical insertion of a piece of chromic gut suture beneath the infraorbital nerve creates a better outcome and a more functionally relevant trigeminal neuropathic pain model that is validated for mice in these studies.

## Results and discussion

### Infraorbital nerve gross anatomy and histology

**Figure 1:**
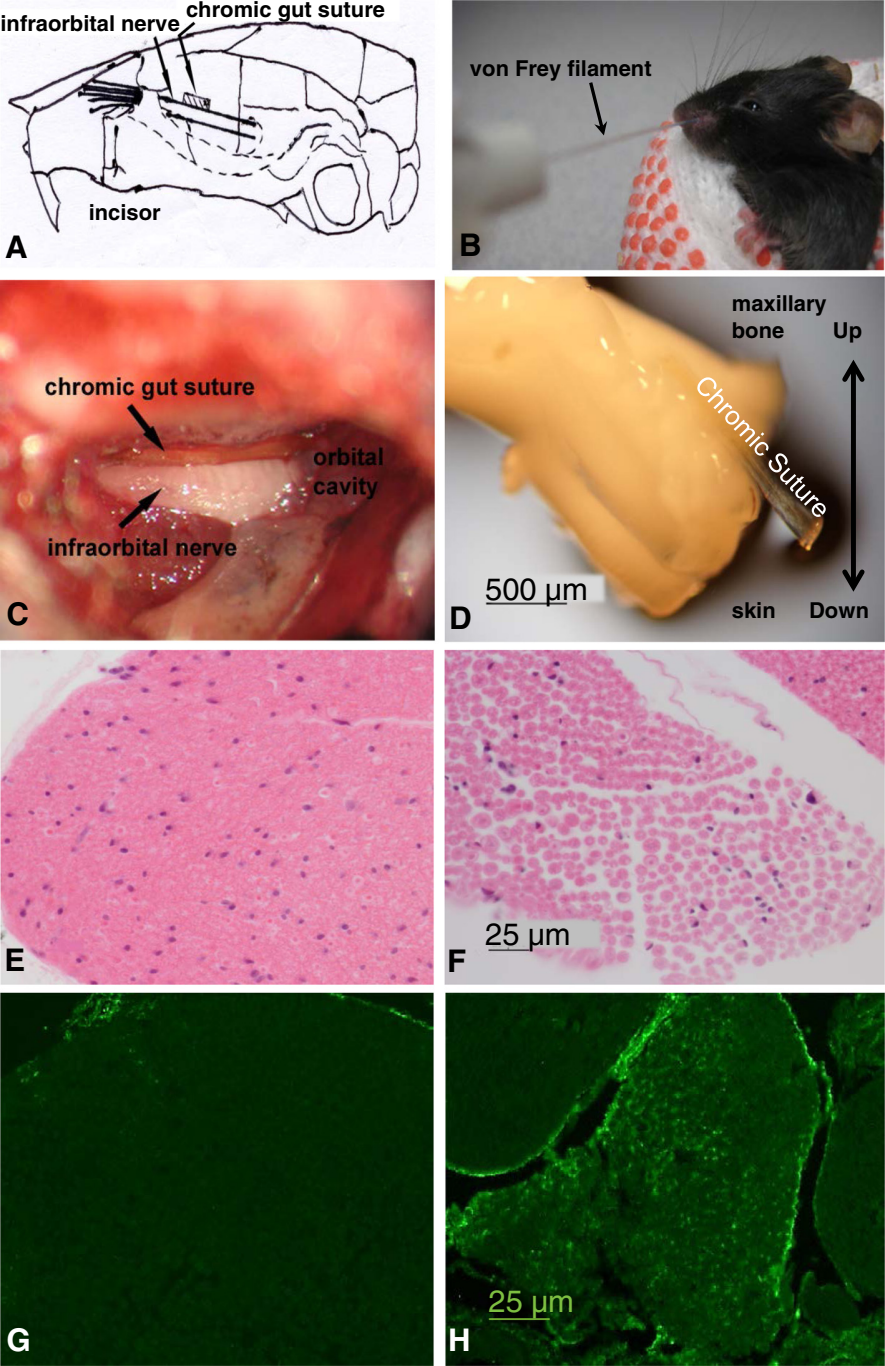
**Chromic gut suture placement and histological evidence of injury at the site of TIC. (A)** Schematic illustration of the chromic gut suture placement between the infraorbital nerve and the maxillary bone. **(B)** During behavioral testing mice are held in a pair of cotton insulated lab gloves by one experimenter and another experimenter probes the mouse whisker pad with the calibrated von Frey filaments to determine the mechanical threshold. **(C)** A photomicroscopic image taken during surgery shows the anatomical relationship of the 2 mm segment of chromic gut suture inserted between the infraorbital nerve and the maxillary bone in the lower orbital cavity. **(D)** A photomicrograph showing the chromic gut suture adhered onto the infraorbital nerve as dissected at the end of the experimental time course (10 weeks). **(E)** H&E histological staining of a normal axon bundle from an animal in the sham group illustrating normal morphology with minimal spaces between axons and intact axonal myelin sheaths. **(F)** The histology of the infraorbital nerve at the site adjacent to the chromic gut suture indicates there was no overt damage to the nerve axons. The spaces between axons are enlarged indicating edema. The axonal myelin sheaths were intact. **(G)** Immunofluorescence for activated macrophage marker, CD68, was not detected in the infraorbital nerves from the sham group mice. **(H)** CD68 immunoreactive was evident throughout the axonal fascicles adjacent to the chromic gut suture indicating inflammatory infiltration.

Anatomical, histological and behavioral confirmation was sought that the chromic gut suture placed between the infraorbital nerve and the maxillary bone was the source of whisker pad hypersensitivity, and that mice in the sham group did not develop hypersensitivity after only surgical exposure of the infraorbital nerve (Figure 1A-D). Detailed description of the surgical method is provided in the Methods. At the end of study (week 10 after TIC nerve trauma), the infraorbital nerve was prepared for macro and microphotographic documentation. An infraorbital nerve with the chromic gut suture still adhered is shown in Figure 1D. The photo indicates that the chromic gut suture remains through this time frame maintaining the chemical-toxic stimulation of the infraorbital nerve and had not been resorbed. Careful handling of the nerve during paraffin embedding maintained the orientation of the infraorbital nerve and adjacent chromic gut suture in the cross sections. Sections were stained with hematoxylin/eosin (H&E) for histological analysis and for immunohistochemical localization of anti-CD68 antibody to detect inflammation (Figure 1E-H). Microscopy detected no significant gross anatomical damage to the infraorbital nerves. H&E staining revealed nerve edema at the chromic gut suture contact site evidenced by enlarged spaces between the axons (Figure 1E, F). The nerves from the sham group were normal and had minimal space between axons and fascicles. The CD68 immunoreactivity in the nerve trunk, a marker for activated macrophages, indicated a significant accumulation of immune cell infiltration at the site of chromic gut suture attachment 10 weeks after surgery (Figure 1G, H).

### Mechanical allodynia after TIC nerve trauma involvement of the infraorbital branch

**Figure 2:**
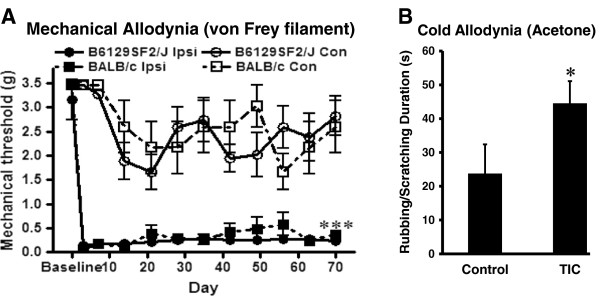
**Lowered mechanical threshold of whisker pad after induction of TIC. (A)** Mechanical threshold was determined for the whisker pad with von Frey filaments before and after infraorbital nerve traumatic compression on day 3 and 7 in the first week, and once a week for the subsequent 9 weeks. The data presented is the 50% mechanical threshold in gram force. Mice had statistically significant decreases in mechanical threshold 3 days after surgical placement of chromic gut suture compared to the sham operated group as well as to the contralateral side, (one-way ANOVA, ***p < 0.001). The continuous mechanical allodynia persisted for 10 weeks. **(B)** Administration of acetone onto the ipsilateral whisker pad elicits a cold allodynia-like response (4 wk). The mice with TIC injury had an increased rubbing/scratching duration time compared to the control mice. *p < 0.05.

Alteration in responses to sensory stimulation was determined on the whisker pad, the receptive field for the infraorbital nerve (Figure 1B). Von Frey filaments were applied to the whisker pad on both ipsilateral and contralateral sides to detect mechanical thresholds on day 3 and 7 in the first week and once per week for 10 weeks. Two mouse strains were tested to determine the validity of this method (Figure 2A). The TIC model mice had detectable mechanical allodynia on the ipsilateral whisker pad 3 days after the surgery. The statistically significant decrease in mechanical threshold compared with the sham group (n = 11 in B6129SF2/J and 5 in BALB/c), persisted more than 10 weeks (0.23 ± 0.10g *vs.* 3.15 ± 1.37g in B6129SF2/J, n = 11, *p* < 0.001 and 0.37 ± 0.16g *vs.* 3.47 ± 0.00g in BALB/c, n = 5, *p* < 0.001). This demonstrates that chromic gut suture causes chronic trigeminal neuropathic pain related behavior continuous for over 10 weeks in mice which has in fact continued through week 14 in another preliminary study (not shown).

### Cold allodynia after TIC nerve trauma

At 4 weeks after induction of TIC, cold-evoked response was assessed with the acetone test. Following topical administration of 20 μl of 90% acetone onto the ipsiateral side whisker pad skin, mice exhibited an immediate grooming behavior focused to the site of application of the cold stimulus (Figure 2B). The acetone produced an allodynia-like response evidenced by a significant increase in the duration of the rubbing/scratching behavior (44.55 ± 6.5s, n = 4) in mice with TIC nerve injury compared to the naïve control group (23.76 ± 8.6 s, n = 8, *p* < 0.05). A subsequent test at 10 weeks revealed no difference between groups.

### Detection of neuronal injury or microglial activation in the trigeminal ganglia and the spinal trigeminal nucleus

**Figure 3:**
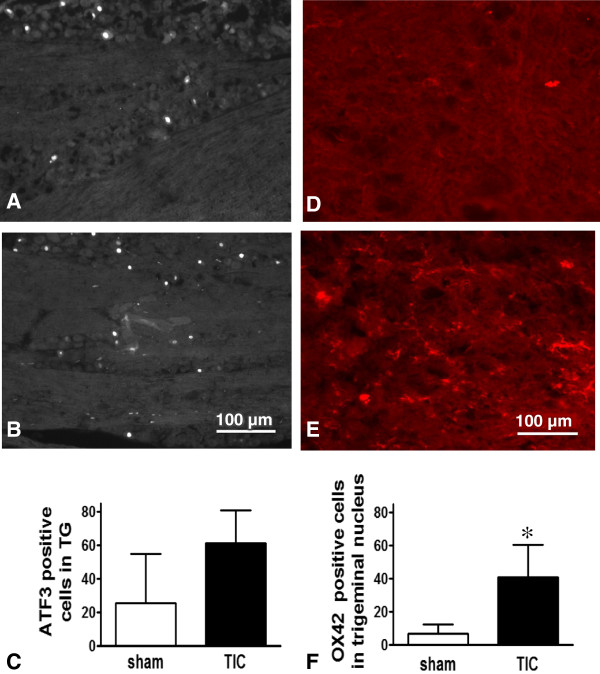
**Neuronal injury marker ATF3 in trigeminal ganglia neurons and microglial activation marker OX42 in spinal trigeminal nucleus. (A)** ATF3 immunoreactivity in trigeminal ganglia neurons trended toward an increase in the primary afferent nerve neurons innervating the whisker pad of mice in the nerve trauma group. Some ATF3 was also observed in the trigeminal ganglia of the sham group. **(B)** Histogram showing ATF3 immunofluorescence increases moderately in week 10 after TIC nerve trauma. **(C)** Histogram showing cells in trigeminal ganglia positively stained for ATF3. There was an increase in ATF3 after infraorbital nerve injury but the increase was not significant. **(D)** OX42 immunohistochemistry in the spinal trigeminal nucleus identified only background levels of staining in the sham group. **(E)** Microglial activation was evident in the spinal trigeminal nucleus after infraorbital nerve trauma at the end of the 10 week experimental time course. **(F)** Bar graph showing the statistically significant increase in OX42 positive cells in the trigeminal nucleus after infraorbital TIC nerve trauma. *p < 0.05.

Trigeminal ganglia (TG) neurons were stained with the neuronal injury marker, ATF3 a member of the ATF/CREB family of transcription factors, to determine the extent of their injury stress response (Figure 3A-C). In week 10 after chromic gut suture placement, there was moderate increase in ATF3 expression in the TG neurons although the increase did not reach statistical significance (61.33 ± 11.26 *vs.* 25.67 ± 16.76, n = 3). ATF3 immunoreactivity was expressed in the nuclei of the TG neurons. There was minimal ATF3 immuno-positive cell staining of the TG in the group receiving sham surgery and in the contralateral TG of the group with infraorbital nerve injury.

Medullary brainstem sections were stained with anti-OX42 antibody to identify activated microglia in the spinal trigeminal nucleus (Figure 3D-F). A statistically significant increase in OX42 immunoreactivity was identified in non-neuronal structures (40.67 ± 11.29 *vs.* 7 ± 5.19, n = 3, *p* < 0.05) at the end of experiment. There were a minimal number of cells expressing OX42 in the trigeminal nucleus of the sham operated group either on the ipsilateral or contralateral side, or on the contralateral side of the group with the TIC model.

### Reversal of mechanical allodynia

Reversal of mechanical allodynia was tested using pharmacological drugs which have been shown to be effective of alleviating nerve injury induced pain. Microglial activation inhibitor, minocycline (30 mg/kg), P2X7 antagonist, A438079 (10 mg/kg) and p38 inhibitor, SB203580 (50 μg/kg) were injected intraperitoneally in the mice of the injured and the sham groups in week 8, 9 and 10 after surgery respectively. Mechanical threshold in both sides of the whisker pads was tested before and at 4 time points within the subsequent 6 hours (30 min, 1 h, 3 h and 6 h) after injection of A438079 and SB203580 and at 1 hour after minocycline injection.

### Minocycline

**Figure 4:**
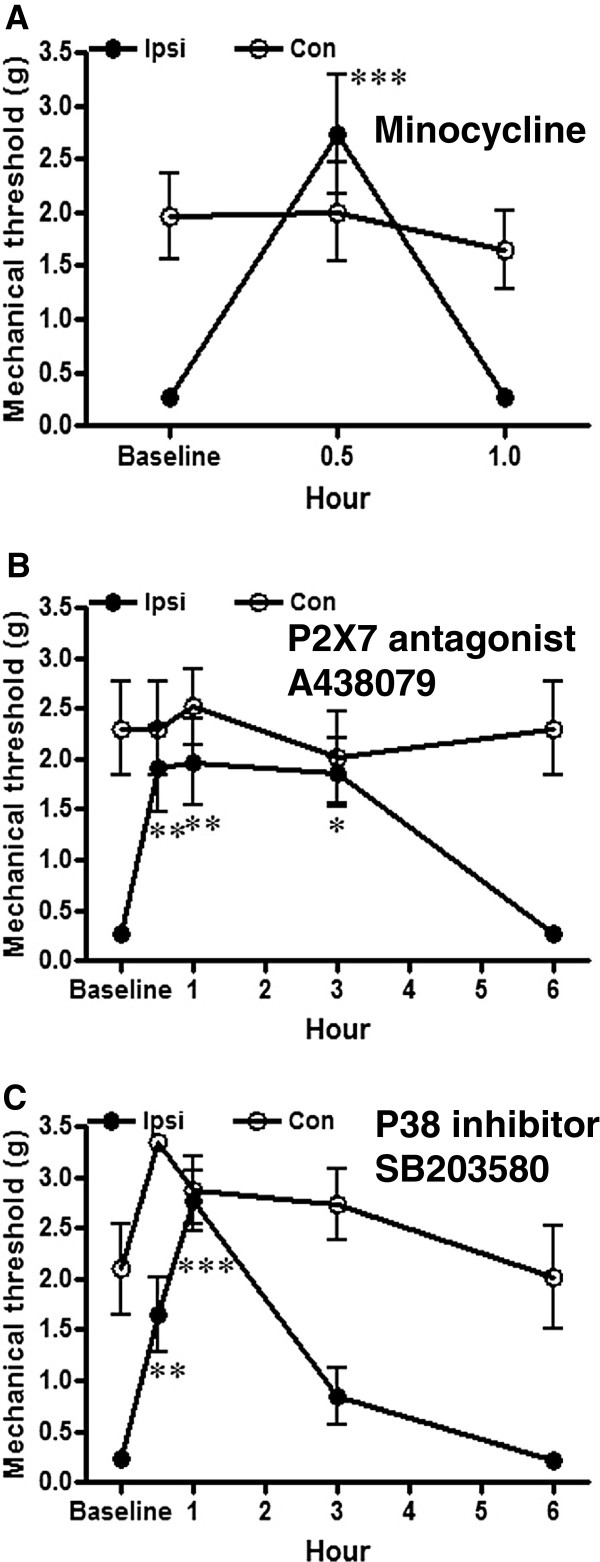
**(A) Alleviation of mechanical allodynia by microglial activation inhibitor, minocycline.** Mechanical threshold was tested before and after intraperitoneal (i.p.) injection of minocycline. The mechanical threshold was increased by minocycline (30 mg/kg, i.p.) 30 min after administration and the effect was diminished within 1 hour. ***p < 0.001. **(B)** P2X7 antagonist, A438079 eliminated mechanical allodynia caused by TIC nerve trauma. Whisker pad mechanical threshold was tested before and after intraperitoneal injection of A438079. The mechanical allodynia was reduced by A438029 (10 mg/kg, i.p.) as early as 30 min after injection. The analgesic effect reached peak at 1h and diminished at 3 h. **p < 0.01, ***p < 0.001. **(C)** P38 inhibitor, SB203580 reversed the TIC nerve trauma induced mechanical allodynia. Mechanical threshold in the whisker pad was tested before and after intraperitoneal injection of SB203580. The mechanical allodynia was reduced by SB203580 (50 μg/kg, i.p.) starting at 30 min. The maximal analgesic effect persisted for 3 hours. *p < 0.05, **p < 0.01.

Mechanical allodynia had been confirmed before the drug administration (Figure 4A). Minocycline alleviated mechanical allodynia in mice with TIC nerve trauma within 1 hour. The minocycline reversal effect reached peak at 0.5 h (2.74 ± 1.38 g *vs.* 0.26 ± 0.04 g, *p* < 0.001, n = 6) and the effect was diminished at 1 h. There was no threshold change on contralateral whisker pad after minocycline administration.

### P2X7 antagonist, A438079

The inhibitory effect of A438079 on mechanical allodynia was evident at 0.5 h and reached a peak at 1 h (1.65 ± 0.89 g and 2.77 ± 0.73 *vs.* 0.23 ± 0.03g, *p* < 0.01 and *p* < 0.001, n = 6) (Figure 4B). Although the mechanical threshold was still elevated at 3 h, there was no statistically significant difference at this time point. The mechanical threshold had returned to the pre-drug level at 6 h. A438079 had no effect on the mechanical threshold on the contralateral side.

### p38 inhibitor SB203580

Mechanical threshold of the affected whisker pad increased rapidly after SB203580 administration and the effect reached a peak at 0.5 h (1.91 ± 1.05 g *vs.* 0.27 ± 0.07 g, *p* < 0.01, n = 6) (Figure 4C). The elevation of mechanical threshold plateau in whisker pad persisted 3 hours (1.97 ± 1.05 g *p* < 0. 01 at 1 h and 1.86 ± 0.82 g, *p* < 0.05 at 3 h, n = 6). The effect diminished at 6 h. The SB203580 did not affect mechanical threshold of the contralateral whisker pad.

### Continuous pain related behaviors induced in the mouse TIC model

Many methods have been used to assess dysfunctional responses related to orofacial pain after nerve injury, including spontaneous pain behaviors and evoked mechanical and thermal stimulation. Among the orofacial neuropathic pain models described for mice, most have focused on the infraorbital nerve with the exception of one study using mental nerve ligation. Although the mental nerve tight ligation model was reportedly more relevant to clinical sensory impairments, the threshold for mechanical touch is increased rather than decreased, which is opposite of the TIC model and reports given by most clinical orofacial pain patients [7]. Other methodologies have been adapted to partially damage the infraorbital nerve to produce mechanical allodynia on the mouse whisker pad. The 7-0 silk suture tight ligation of half to one third of the infraorbital nerve approached surgically through the orbital cavity produces mechanical allodynia lasting as long as 4 weeks [5]. While the partial ligation and partial transection of the infraorbital nerve introduces increased facial grooming and mechanical allodynia, loose ligation of the infraorbital nerve with two 4-0 silk ligatures also induces thermal hyperalgesia in mice [4,6,12].

We report here a novel chronic orofacial neuropathic pain model inducing continuous pain related mechanical allodynia responses in mice. The method has several improvements over other previously published models. Mechanical allodynia is evident on the whisker pad in 100% of the mice 3 days after surgery and persists for at least 10 weeks. Cold allodynia was evident at a timepoint 4 weeks after induction of the TIC model. Given the difficulties in performing orofacial surgery in mice due to their size, the narrow margin between effective and lethal doses of anesthesia, and the potential for excessive bleeding from the orbital cavity, the method described for placement of chromic gut suture between the infraorbital nerve and maxillary bone is a simpler, rapid, and more efficient procedure for reliable induction of a chronic, continuous orofacial neuropathic pain model in mice.

With our modified behavioral testing method, we are able to detect the chronic orofacial nociception changes reliably in the mice through 10–14 weeks. The holding strategy is critical for successful behavioral testing in mice which are more difficult to manage than rats. Gently restraining the mice between two hands with insulating cotton gloves provides a more comfortable habituation and testing environment, as well as minimizes stress for the mice. This method generally provides successful acquisition of the data and appears to be better than other reported methods for testing the orofacial area which put mice in abnormal and stressful positions, including either pulling the mouse’s tail or grasping the mouse by the back [7,13]. The mechanical allodynia is produced in two mouse strains indicating that the model has general applicability.

### Minimal injuries at the affected infraorbital nerve site

As shown, chromic gut suture remains along the infraorbital nerve through the entire experimental time course. There is a mild compression of the nerve caused by the placement of the 2 mm chromic gut suture material between the maxillary bone and the infraorbital nerve. No obvious nerve damage was noted with H&E staining. Compared to the histological appearance of normal nerve bundles, enlarged spaces were evident between axons at the site where the chromic gut suture was adhered, likely caused by the edema and accompanying inflammatory cell infiltration. Chemical nerve injury with other methods may likewise be good regimes as trigeminal pain models. For example, when CFA oxycel was applied to the surface of the infraorbital nerve facial grooming is induced indicative of on-going nociception caused by the edema at the affected nerve site [14]. In another inflammatory model, injection of coral snake venom into the infraorbital nerve produced persistent ipsilateral mechanical allodynia for 60 days [15]. Many models of inflammation that induce nerve injury have been applied to other nerves. Sciatic nerve immune cell accumulation at the site of CFA soaked gauze was shown by immunofluorescence of CD11b (OX42), a marker for activated macrophages. Thermal hyperalgesia and mechanical allodynia were both noted with rat hindpaw testing [16]. Our histology results showed that there is nerve inflammation at the site of the chromic gut suture placement. Along with the edema in the axon bundle, CD68, another marker for macrophages or microglia was positively stained in the spaces among the axons on the serial infraorbital nerve sections. Maves et al. [8] tested the contribution of the chemical toxicity produced by the chromic gut suture on sensory nerve dysfunction after placing different suture materials adjacent to the sciatic nerve. Immune-mediated response, axonal compression and injury induced by nerve ligation have also been confirmed by other researchers [17,18]. Constriction of sciatic nerve with chromic gut suture model has been widely used since 1988 [3]. This nerve injury is reportedly due to both the constricted epineurial vasculature and the response to the chromic gut suture. In our previous study using chromic gut suture ligation of the infraorbital nerve in rats [19], a statistically significant decrease in axonal numbers compared to controls was evident.

### Inflammatory immunoreactivity in trigeminal ganglia neurons and spinal trigeminal nucleus

The present results indicate that the chromic gut suture caused partial nerve compression and the toxic chemicals released locally produced irritation and mild inflammatory response in our mouse model sufficient to produce continuous mechanical allodynia for at least 10 weeks. ATF3 is significantly up-regulated for several weeks in the affected TG neurons, appearing as early as day 3 after nerve injury in both rats and mice after severely damaging the nerve by constrictive ligation or partial tight ligation [5,20]. A previous study in our lab found ATF3 expression is also maintained through 10 weeks in the affected TG neurons after constrictive infraorbital injury in rats [19]. Our findings are similar to observations by Tsuzuki et al. [21] who found that ATF3 can be expressed in the TG neurons at least 4 weeks after infraorbital nerve transection. Mechanical allodynia induced in our mouse model is a result of central sensitization caused by the persistent immune reaction and sensory dysfunction created by the physical compression of the infraorbital nerve.

We further investigated whether infraorbital nerve compression induced by chromic gut suture would trigger glial activation in the spinal trigeminal nucleus as widely reported by other types of neuropathic pain. Microglial activation indicated by up-regulation of OX-42 is commonly used as a biomarker for detection of neuropathic injury in either the spinal cord or the spinal trigeminal nucleus [22-24]. Central microglial response can be activated by either neuronal injury or peripheral nerve inflammation [25]. The increased OX42 positive immunoreactivity evident in the spinal trigeminal nucleus at 10 weeks indicates chronic nerve inflammation and microglial activation. The spinal trigeminal nucleus is the site of synaptic contact between the trigeminal nerve endings and the second order neurons in the sensory transmission pathway relaying the information about pain to higher brain centers. Long-term microglial activation has not yet been reported in any other mouse trigeminal neuropathic pain models. Our model combines mild nerve compression and inflammation as well as central microglial activation at the level of the second order spinal trigeminal relay neurons providing adequate histological evidence of dysfunctional sensory transmission. As has been suggested by others for spinally mediated sensory disturbance, trigeminal nucleus microglial activation is also a causal factor contributing to the mechanical allodynia at the whisker pad observed after nerve injury.

### Pharmacological reduction of mechanical allodynia in the TIC model

Minocycline, microglial activation inhibitor, systemically administered to the mice with TIC provided an inhibitory effect persisting 30 min. Although studies have shown inhibition of microglial activation reverses mechanical allodynia after multiple doses of minocycline [10,26], an acute effect was reported after ventral posterolateral thalamic injections for sciatic constrictive injury [27]. It is not clear if the inhibitory effect of minocycline in the present study is a direct effect in the trigeminal nucleus or at other sites. P2X7 is one of the receptors involved in oxidative stress, microglial activation, and mediated release of inflammatory molecules from glial cells [28,29]. The P2X7 antagonist has been shown to alleviate thermal hyperalgesia induced by complete Freund’s adjuvant through interleukin 1β release from microglial cells [30]. In the mouse TIC model characterized here, application of A438079 had a longer anti-hyperalgesic effect than minocycline suggesting that specific inhibition of microglial activation could better reverse hypersensitization. Likewise in the pharmacological data shown, inhibition of the MAP Kinase signaling pathway molecule p38 diminished mechanical allodynia induced by the TIC model. As a MAP kinase downstream molecule, p38 is activated and also involved in microglial activation induced by peripheral nerve injury [11,31]. The anti-allodynic effects may be due to reduced microglial activation or other downstream signaling events [32,33].

## Conclusions

To summarize our results, the continuous pain related behaviors, aberrant nerve histology and inflammatory biomarker immunoreactivity in the spinal trigeminal nucleus establish the TIC method as a new stable and reliable mouse model of trigeminal inflammatory compression. The model is relevant to clinical type 2 orofacial chronic neuropathic pain with continuous burning pain. It is unknown if the method reproduces the lancinating bursts of nerve activity that are also a characteristic of this orofacial pain condition (type I, tic doloreux), but further study may find evidence for this with nerve recordings. The pharmacological results provide further evidence for the alleviation of hypersensitivity in the TIC model with drugs that have been shown to be effective by several others that have explored therapeutic targets in infraorbital nerve constrictive injury or partial ligation models [34-36].

## Materials and methods

### Animals

Male B6129SF2/J and BALB/c mice (The Jackson Laboratory), weighing 20–30 g at the beginning of the study, were accommodated in ventilated animal housing with a reversed 10/14 h dark/light cycle. Experiments were carried out in accordance with the Guidelines established by National Institute of Health (NIH) regarding the care and use of animals for experimental procedures. Protocols were approved by the Institutional Animal Care and Use Committee at the University of Kentucky.

### Chemicals

Microglial activation inhibitor, minocycline (Sigma, St. Louis, MO), P2X7 antagonist, A438079 and p38 inhibitor, SB203580 (Tocris, Bristol, BS11 0QL, UK) were dissolved in saline before injection.

### Surgery

Mice were anesthetized with sodium pentobarbital (70 mg/kg, i.p.), and all surgeries were performed in sterile conditions under a surgical microscope. The hair on the top of the head was shaved and the mouse placed in a stereotaxic frame. Ophthalmic cream was applied to the corner of both eyes to prevent drying damage. An anterior-posterior 15 mm skin incision was made at midline of the head. The infraorbital muscle was gently dissected from the bone until the orbit could be gently retracted. A piece of gel-foam or a tiny cotton ball was packed into orbital cavity to minimize bleeding. The infraorbital nerve can be seen approximately 5 mm deep within the orbital cavity, lying in the infraorbital bony fissure. The infraorbital nerve was dissected free from the bone at its most rostral extent in the orbital cavity, and a single 2 mm length of chromic gut suture (6-0) was inserted between the infraorbital nerve and the maxillary bone (Figure 1A, C). In the sham operation control group, only skin incision and muscle dissection were performed. The nerve was not touched and no chromic gut suture was inserted. All skin incisions were sutured with 5-0 nylon non-absorbable monofilament, and mice were allowed to recover for three days.

### Assessment of mechanical allodynia on the whisker pad and drugs tested

Mechanical sensitivity of the whisker pad, the infraorbital nerve receptive field, was measured with a series of 8 von Frey fiber filament (0.008 g (1.65); 0.02 g (2.36); 0.07 g (2.83); 0.16 g (3.22); 0.4 g (3.61); 1.0 g (4.08); 2.0 g (4.31); 6.0 g (4.74); Stoelting, Wood Dale, IL) by modified up-down method. Mice were handled several times before experiments. One experimenter held the mouse with two hands in insulating cotton gloves until the animal was calm. Animal moved freely in the holder’s hands with its head exposed as shown in Figure 1B. During testing, one experimenter slightly restrains the mouse in their hands so that another experimenter could accurately apply the von Frey filament onto the center of the mouse whisker pad, both ipsilateral and contralateral to the surgery site. For consistency of results, each filament was applied five times at intervals of a few seconds. If head withdrawal was observed at least three times after probing with a filament, the mouse was considered responsive to that filament according to the up-down method [37,38]. For this approach, whenever a positive response to the mechanical stimulus occurred, the next weaker von Frey filament was applied. If no positive response is evoked, the next stronger filament was applied. Testing proceeded in this manner until four fibers applied after the first one successfully caused positive responses. This allowed estimation of the 50% mechanical withdrawal threshold (in gram) using a curve-fitting algorithm. The mechanical thresholds on the whisker pads of both sides were measured on day 3 and 7 in the first week and then once a week for 10 weeks after surgery. To test effects of drugs on the behavioral changes, mechanical allodynia was confirmed in the mice after induction of TIC nerve trauma in the late weeks of experimental period. Minocycline, A438079 and SB203580 were each injected into mice intraperitoneally. The behavioral changes were tested at 0.5, 1, 3 and 6 h after drug administration except minocycline which had a 1 h testing duration. To conserve animals, mice were tested with all drugs but with only one drug per week allowing recovery time before another drug was tested.

### Assessment of cold allodynia on the whisker pad

Mice (C57Bl/6) were acclimated in the see-through plastic observation chamber (28 × 17.5 × 12.5 cm) with 1 mirrored side in an isolated room with constant “white noise” for ten minutes. After the acclimation period, 20 μl of 90% acetone was applied to both the control and mice with TIC on the ipsilateral side of the whisker pad with a customized 25-gauge needle (blunt and slightly bent) attached to a 50 μl microsyringe (Hamilton, Reno, NV). Special care was taken to avoid acetone leakage near the ocular surface or the nose [39]. A digital camcorder located 0.5 m from the chamber with an unobstructed view was used to record animal spontaneous nocifensive behavior for 5 minutes. The camcorder was linked to a computer recording program for offline data analysis (Logitech Image Studio; Logitech, Fremont, CA). The chamber was washed with a detergent/disinfectant and dried between animals. The nocifensive behavior evaluated in this study was asymmetric orofacial grooming, i.e. rubbing and scratching focused on the whisker pad and executed with the ipsilateral forepaw. The videos were analyzed and only the first two minutes of behavior after acetone administration were recorded for duration of rubbing/scratching events.

### Morphological study methods

#### Aldehyde fixation

At the end of the study (week 10 after nerve trauma), mice were anesthetized with isoflurane and perfused transcardially with heparinized saline followed by 4% ice-cold paraformaldehyde in 0.1 M phosphate buffer solution (PB, pH 7.4).

#### Paraffin embedding

Infraorbital nerves were dissected out and placed in the same fixative solution at 4°C overnight. Samples were switched to 70% ethanol, photographed, dehydrated through graded ethanol, and embedded in paraffin. Infraorbital nerve tissue sections were cut (5 μm), mounted onto glass slides (Super Frost Plus, VWR, Radnor, PA), deparaffinized (Citrisolv, Fisher), rehydrated with graded ethanol, and rinsed in tap water.

#### Hematoxylin and Eosin (H&E) staining

Hydrated slides were immersed in 0.1% hematoxylin for 1–3 min, washed in tap water, then immersed in 0.1% eosin for 1 min, and dehydrated through graded ethanol. Finally sections were coverslipped with Permount (Fisher, Pittsburgh, PA).

#### Immunofluorescent staining

The medulla and the TG were dissected after fixative perfusion and post fixed for 4 hours. The tissues were switched to 30% sucrose in PB for 18–24 hours and embedded into O.C.T. compound (Tissue-Tek, Sakura, Torrance, CA). The tissue blocks were cryosectioned (10 μm) and mounted onto Super Plus glass slides. The nerve and TG as well as the medullary brainstem sections containing the spinal trigeminal nuclei were washed with 0.1 M phosphate buffered saline (PBS, pH7.4) and blocked with 3% normal goat serum (30 min, RT). Sections were incubated overnight at room temperature with goat anti-CD68 (1:100); mouse anti-OX42 (1:1000, Abcam, Cambridge, MA), or rabbit anti-activating transcription factor 3 (ATF3) (1:200, Santa Cruz, Santa Cruz, CA) antibodies for immunolocalization of biomarkers for monocyte/macrophage invasion or injured nerves, respectively. Subsequently, sections were incubated with secondary antibodies (Alexa Fluor 488 donkey anti-goat; Alexa Fluor 594 goat anti-rabbit and mouse (1:1000, 1 h, Invitrogen, Grand Island, NY). Sections were coverslipped with anti-fade, glycerol based mounting media with/without DAPI (Vector Laboratories, Burlingame, CA) and visualized using a Nikon E1000 microscope (Nikon Instruments, Inc., Melville, NY) equipped with MetaVue and Act-1 Programs.

#### Image analysis

Five images from each animal were digitally captured was analyzed using the Metamoph off line analysis program. Mean fluorescent intensities in different experimental groups were plotted and compared.

### Statistical analysis

The Prism 4 statistical program was used for data analysis (Graph Pad Software, Inc., La Jolla, CA). All data were expressed as mean ± SD. The weekly behavioral changes after nerve injury among the four groups for the ipsilateral and contralateral sides (10 weeks) were analyzed by *one-way ANOVA* followed by Tukey's Multiple Comparison Post hoc testing. A *p* ≤ 0.05 was considered significant. Histological analyses were done using *Student’s t-test* with p < 0.05 considered significant.

## Abbreviations

CCI: Chronic constriction injury; H&E: Hematoxylin/eosin; PBS: Phosphate buffered saline; TIC: Trigeminal inflammatory compression; TG: Trigeminal ganglia.

## Competing interest

None of the authors have any financial or other relationships that might lead to a conflict of interests.

## Authors’ contributions

All authors have read and approved the final manuscript. FM, LZ and KNW participated in the conception, design, and interpretation of the study. FM, LZ and DL carried out the experiments. FM wrote the draft of the manuscript and prepared the figures. LZ edited the text and figures. KNW edited the text and figures for the final submission.

## References

[B1] De Leeuw R Episodic and Continuous Neuropathic Pain Orofacial Pain 2008 4 Hanover Park IL: Quintessence Pub Co, Inc 83 99

[B2] Vos BP Strassman AM Maciewicz RJ Behavioral evidence of trigeminal neuropathic pain following chronic constriction injury to the rat's infraorbital nerve J Neurosci 1994 14 2708 2723 8182437 10.1523/JNEUROSCI.14-05-02708.1994 PMC6577477

[B3] Bennett GJ Xie YK A peripheral mononeuropathy in rat that produces disorders of pain sensation like those seen in man Pain 1988 33 87 107 10.1016/0304-3959(88)90209-6 2837713

[B4] Luiz AP Schroeder SD Chichorro JG Calixto JB Zampronio AR Rae GA Kinin B(1) and B(2) receptors contribute to orofacial heat hyperalgesia induced by infraorbital nerve constriction injury in mice and rats Neuropeptides 2010 44 87 92 10.1016/j.npep.2009.10.005 19914714

[B5] Xu M Aita M Chavkin C Partial infraorbital nerve ligation as a model of trigeminal nerve injury in the mouse: behavioral, neural, and glial reactions J Pain 2008 9 1036 1048 10.1016/j.jpain.2008.06.006 18708302 PMC2632609

[B6] Miyamoto M Tsuboi Y Takamiya K Huganir RL Kondo M Shinoda M Oi Y Iwata K Involvement of GluR2 and GluR3 subunit C-termini in the trigeminal spinal subnucleus caudalis and C1-C2 neurons in trigeminal neuropathic pain Neurosci Lett 2011 491 8 12 10.1016/j.neulet.2010.12.060 21215292 PMC3130337

[B7] Seino H Seo K Maeda T Someya G Behavioural and histological observations of sensory impairment caused by tight ligation of the trigeminal nerve in mice J Neurosci Methods 2009 181 67 72 10.1016/j.jneumeth.2009.04.020 19409417

[B8] Maves TJ Pechman PS Gebhart GF Meller ST Possible chemical contribution from chromic gut sutures produces disorders of pain sensation like those seen in man Pain 1993 54 57 69 10.1016/0304-3959(93)90100-4 8378104

[B9] Chessell IP Hatcher JP Bountra C Michel AD Hughes JP Green P Egerton J Murfin M Richardson J Peck WL Disruption of the P2X7 purinoceptor gene abolishes chronic inflammatory and neuropathic pain Pain 2005 114 386 396 10.1016/j.pain.2005.01.002 15777864

[B10] Guasti L Richardson D Jhaveri M Eldeeb K Barrett D Elphick MR Alexander SP Kendall D Michael GJ Chapman V Minocycline treatment inhibits microglial activation and alters spinal levels of endocannabinoids in a rat model of neuropathic pain Mol Pain 2009 5 35 10.1186/1744-8069-5-35 19570201 PMC2719614

[B11] Terayama R Omura S Fujisawa N Yamaai T Ichikawa H Sugimoto T Activation of microglia and p38 mitogen-activated protein kinase in the dorsal column nucleus contributes to tactile allodynia following peripheral nerve injury Neuroscience 2008 153 1245 1255 10.1016/j.neuroscience.2008.03.041 18440713

[B12] Alvarez P Brun A Labertrandie A Lopez J Correa A Constandil L Hernandez A Pelissier T Antihyperalgesic effects of clomipramine and tramadol in a model of posttraumatic trigeminal neuropathic pain in mice J Orofac Pain 2011 25 354 363 22247931

[B13] Krzyzanowska A Pittolo S Cabrerizo M Sanchez-Lopez J Krishnasamy S Venero C Avendano C Assessing nociceptive sensitivity in mouse models of inflammatory and neuropathic trigeminal pain J Neurosci Methods 2011 201 46 54 10.1016/j.jneumeth.2011.07.006 21782847

[B14] Benoliel R Wilensky A Tal M Eliav E Application of a pro-inflammatory agent to the orbital portion of the rat infraorbital nerve induces changes indicative of ongoing trigeminal pain Pain 2002 99 567 578 10.1016/S0304-3959(02)00272-5 12406533

[B15] An JX He Y Qian XY Wu JP Xie YK Guo QL Williams JP Cope DK A new animal model of trigeminal neuralgia produced by administration of cobra venom to the infraorbital nerve in the rat Anesth Analg 2011 113 652 656 21778333 10.1213/ANE.0b013e3182245add

[B16] Amaya F Samad TA Barrett L Broom DC Woolf CJ Periganglionic inflammation elicits a distally radiating pain hypersensitivity by promoting COX-2 induction in the dorsal root ganglion Pain 2009 142 59 67 10.1016/j.pain.2008.11.013 19135800 PMC2755568

[B17] Campbell JN Meyer RA Mechanisms of neuropathic pain Neuron 2006 52 77 92 10.1016/j.neuron.2006.09.021 17015228 PMC1810425

[B18] Kawakami M Weinstein JN Chatani K Spratt KF Meller ST Gebhart GF Experimental lumbar radiculopathy. Behavioral and histologic changes in a model of radicular pain after spinal nerve root irritation with chromic gut ligatures in the rat Spine (Phila Pa 1976) 1994 19 1795 1802 10.1097/00007632-199408150-00002 7973977

[B19] Ma F Zhang L Westlund KN Trigeminal nerve injury ErbB3/ErbB2 promotes mechanical hypersensitivity Anesthesiology 2012 117 381 388 10.1097/ALN.0b013e3182604b2b 22705569 PMC3406246

[B20] Kernisant M Gear RW Jasmin L Vit JP Ohara PT Chronic constriction injury of the infraorbital nerve in the rat using modified syringe needle J Neurosci Methods 2008 172 43 47 10.1016/j.jneumeth.2008.04.013 18501433 PMC2497464

[B21] Tsuzuki K Kondo E Fukuoka T Yi D Tsujino H Sakagami M Noguchi K Differential regulation of P2X(3) mRNA expression by peripheral nerve injury in intact and injured neurons in the rat sensory ganglia Pain 2001 91 351 360 10.1016/S0304-3959(00)00456-5 11275393

[B22] Aita M Byers MR Chavkin C Xu M Trigeminal injury causes kappa opioid-dependent allodynic, glial and immune cell responses in mice Mol Pain 2010 6 8 10.1186/1744-8069-6-8 20109235 PMC2826348

[B23] Gao YJ Ji RR Chemokines, neuronal-glial interactions, and central processing of neuropathic pain Pharmacol Ther 2010 126 56 68 10.1016/j.pharmthera.2010.01.002 20117131 PMC2839017

[B24] Piao ZG Cho IH Park CK Hong JP Choi SY Lee SJ Lee S Park K Kim JS Oh SB Activation of glia and microglial p38 MAPK in medullary dorsal horn contributes to tactile hypersensitivity following trigeminal sensory nerve injury Pain 2006 121 219 231 10.1016/j.pain.2005.12.023 16495005

[B25] Roberts J Ossipov MH Porreca F Glial activation in the rostroventromedial medulla promotes descending facilitation to mediate inflammatory hypersensitivity Eur J Neurosci 2009 30 229 241 10.1111/j.1460-9568.2009.06813.x 19614984 PMC5693227

[B26] Chang YW Waxman SG Minocycline attenuates mechanical allodynia and central sensitization following peripheral second-degree burn injury J Pain 2010 11 1146 1154 20418178 10.1016/j.jpain.2010.02.010

[B27] Leblanc BW Zerah ML Kadasi LM Chai N Saab CY Minocycline injection in the ventral posterolateral thalamus reverses microglial reactivity and thermal hyperalgesia secondary to sciatic neuropathy Neurosci Lett 2011 498 138 142 10.1016/j.neulet.2011.04.077 21571034

[B28] Chu YX Zhang Y Zhang YQ Zhao ZQ Involvement of microglial P2X7 receptors and downstream signaling pathways in long-term potentiation of spinal nociceptive responses Brain Behav Immun 2010 24 1176 1189 10.1016/j.bbi.2010.06.001 20554014

[B29] Clark AK Wodarski R Guida F Sasso O Malcangio M Cathepsin S release from primary cultured microglia is regulated by the P2X7 receptor Glia 2010 58 1710 1726 10.1002/glia.21042 20629190

[B30] Honore P Donnelly-Roberts D Namovic M Zhong C Wade C Chandran P Zhu C Carroll W Perez-Medrano A Iwakura Y Jarvis MF The antihyperalgesic activity of a selective P2X7 receptor antagonist, A-839977, is lost in IL-1alphabeta knockout mice Behav Brain Res 2009 204 77 81 10.1016/j.bbr.2009.05.018 19464323

[B31] Suter MR Berta T Gao YJ Decosterd I Ji RR Large A-fiber activity is required for microglial proliferation and p38 MAPK activation in the spinal cord: different effects of resiniferatoxin and bupivacaine on spinal microglial changes after spared nerve injury Mol Pain 2009 5 53 10.1186/1744-8069-5-53 19772627 PMC2759920

[B32] Xu JT Xin WJ Wei XH Wu CY Ge YX Liu YL Zang Y Zhang T Li YY Liu XG p38 activation in uninjured primary afferent neurons and in spinal microglia contributes to the development of neuropathic pain induced by selective motor fiber injury Exp Neurol 2007 204 355 365 10.1016/j.expneurol.2006.11.016 17258708

[B33] Zhuang ZY Kawasaki Y Tan PH Wen YR Huang J Ji RR Role of the CX3CR1/p38 MAPK pathway in spinal microglia for the development of neuropathic pain following nerve injury-induced cleavage of fractalkine Brain Behav Immun 2007 21 642 651 10.1016/j.bbi.2006.11.003 17174525 PMC2084372

[B34] Deseure K Breand S Colpaert FC Curative-like analgesia in a neuropathic pain model: parametric analysis of the dose and the duration of treatment with a high-efficacy 5-HT(1A) receptor agonist Eur J Pharmacol 2007 568 134 141 10.1016/j.ejphar.2007.04.022 17512927

[B35] Kayser V Latremoliere A Hamon M Bourgoin S N-methyl-D-aspartate receptor-mediated modulations of the anti-allodynic effects of 5-HT1B/1D receptor stimulation in a rat model of trigeminal neuropathic pain Eur J Pain 2011 15 451 458 10.1016/j.ejpain.2010.09.012 20965753

[B36] Nakae A Nakai K Yano K Hosokawa K Shibata M Mashimo T The animal model of spinal cord injury as an experimental pain model J Biomed Biotechnol 2011 2011 939023 21436995 10.1155/2011/939023 PMC3062973

[B37] Chaplan SR Bach FW Pogrel JW Chung JM Yaksh TL Quantitative assessment of tactile allodynia in the rat paw J Neurosci Methods 1994 53 55 63 10.1016/0165-0270(94)90144-9 7990513

[B38] Ren K An improved method for assessing mechanical allodynia in the rat Physiol Behav 1999 67 711 716 10.1016/S0031-9384(99)00136-5 10604842

[B39] Constandil L Goich M Hernández A Bourgeais L Cazorla M Hamon M Villanueva L Pelissier T Cyclotraxin-B, a new TrkB antagonist, and glial blockade by propentofylline, equally prevent and reverse cold allodynia induced by BDNF or partial infraorbital nerve constriction in mice J Pain 2012 Jun 13 6 579 589 10.1016/j.jpain.2012.03.008 22560237

